# Functional Connectivity of the Anterior Nucleus of the Thalamus in Pediatric Focal Epilepsy

**DOI:** 10.3389/fneur.2021.670881

**Published:** 2021-08-02

**Authors:** Rory J. Piper, Chayanin Tangwiriyasakul, Elhum A. Shamshiri, Maria Centeno, Xiaosong He, Mark P. Richardson, Martin M. Tisdall, David W. Carmichael

**Affiliations:** ^1^Department of Neurosurgery, John Radcliffe Hospital, Oxford, United Kingdom; ^2^Department of Neurosurgery, Great Ormond Street Hospital for Children, UCL Great Ormond Street Institute of Child Health, London, United Kingdom; ^3^Wellcome EPSRC Centre for Medical Imaging, Department of Biomedical Engineering, King's College London, London, United Kingdom; ^4^Department of Basic and Clinical Neuroscience, Institute of Psychiatry Psychology and Neuroscience, King's College London, London, United Kingdom; ^5^School of Biomedical Engineering and Imaging Sciences, King's College London, London, United Kingdom; ^6^San Francisco Veterans Affairs Health Care System (SFVAHCS), San Francisco, CA, United States; ^7^Department of Psychiatry, University of California, San Francisco, San Francisco, CA, United States; ^8^Sierra Pacific Mental Illness Research Education and Clinical Centers, San Francisco, CA, United States; ^9^Epilepsy Unit, Neurology Department, Hospital Clinic, Barcelona, Spain; ^10^Department of Psychology, University of Science and Technology of China, Hefei, China

**Keywords:** epilepsy, focal epilepsies, childhood epilepsies, deep brain stimulation, functional magnetic resonance imaging, electroencephaloagraphy, connectivity, thalamus

## Abstract

**Objective:** Whilst stimulation of the anterior nucleus of the thalamus has shown efficacy for reducing seizure frequency in adults, alterations in thalamic connectivity have not been explored in children. We tested the hypotheses that (a) the anterior thalamus has increased functional connectivity in children with focal epilepsy, and (b) this alteration in the connectome is a persistent effect of the disease rather than due to transient epileptiform activity.

**Methods:** Data from 35 children (7–18 years) with focal, drug-resistant epilepsy and 20 healthy children (7–17 years) were analyzed. All subjects underwent functional magnetic resonance imaging (fMRI) whilst resting and were simultaneously monitored with scalp electroencephalography (EEG). The fMRI timeseries were extracted for each Automated Anatomical Labeling brain region and thalamic subregion. Graph theory metrics [degree (DC) and eigenvector (EC) centrality] were used to summarize the connectivity profile of the ipsilateral thalamus, and its thalamic parcellations. The effect of interictal epileptiform discharges (IEDs) captured on EEG was used to determine their effect on DC and EC.

**Results:** DC was significantly higher in the anterior nucleus (*p* = 0.04) of the thalamus ipsilateral to the epileptogenic zone in children with epilepsy compared to controls. On exploratory analyses, we similarly found a higher DC in the lateral dorsal nucleus (*p* = 0.02), but not any other thalamic subregion. No differences in EC measures were found between patients and controls. We did not find any significant difference in DC or EC in any thalamic subregion when comparing the results of children with epilepsy before, and after the removal of the effects of IEDs.

**Conclusions:** Our data suggest that the anterior and lateral dorsal nuclei of the thalamus are more highly functionally connected in children with poorly controlled focal epilepsy. We did not detect a convincing change in thalamic connectivity caused by transient epileptiform activity, suggesting that it represents a persistent alteration to network dynamics.

## Introduction

Focal epilepsy is increasingly recognized as a disorder of brain connectivity (1–4), and both structural and functional connectome studies suggest that large-scale network alterations associate with epilepsy (5, 6). The thalamus has been implicated as a major “hub” in epilepsy since it integrates information across multiple functional cortical networks (7). Thalamo-cortical connectivity has long been implicated in the network-based pathogenesis of epileptic seizures (8–10), and has been shown to be a mechanism of interhemispheric seizure propagation, both in generalized (11, 12) and focal epilepsies (13).

Deep brain stimulation (DBS) has therefore become a potential surgical treatment for epilepsy and the SANTE trial in 2010 showed that bilateral stimulation of the anterior nuclei of the thalamus reduced seizure frequency in adults with both medically refractory, temporal lobe, and extratemporal lobe epilepsy (14). Mechanistic understanding of the alterations to thalamic connectivity that may be targeted by this therapy are invaluable for increasing, and potentially predicting efficacy. Thalamic DBS could be therapeutic in children but thus far has not yet been accepted as a therapeutic option to the same degree as in adults. In part this may be explained by the paucity of data on therapeutic outcomes of DBS in this group (15).

There have been a number of human neuroimaging studies in temporal lobe epilepsy (TLE) in adults that examined the significance of the thalamus in the functional and structural connectomes. Magnetic resonance imaging (MRI) currently offers the only non-invasive method of examining the role of thalamic connectivity within the whole-brain connectome. Graph theory has been increasingly used to analyze these structural and functional brain networks to understand the alterations posed by disease states such as epilepsy. Measures such as degree centrality (number of functional connections with other brain regions) and eigenvector centrality (“influence” of a node within a network) have commonly been used.

Studies from functional MRI (fMRI) have shown that the thalamus is a hub of functional connectivity in patients with TLE when compared to controls (16). The study by He et al. (16) demonstrated that the thalami of patients with TLE who were not seizure-free following temporal lobe resection had a higher degree and eigenvector centrality than compared to patients rendered seizure free and healthy controls. Studies of connectivity using diffusion tensor imaging (DTI) have shown findings suggestive of decreased structural connectivity between medial thalamic and temporal regions. Whilst this may seem paradoxical to findings from functional studies, this still strengthens the association of the abnormal connectivity between thalamus and the epileptogenic zone (17, 18). What is not clear from these imaging studies, however, is whether this altered thalamic connectivity is a result of the persistent effect of epilepsy or is instead due to transient interictal epileptiform activity. Interictal epileptiform discharges (IEDs) have been associated with significant thalamic or basal ganglia involvement in children with focal epilepsy (19, 20). This raises a question as to whether the differences in the functional connectivity seen in other studies may be driven by the transient epileptiform activity.

There has been notably less work on establishing the significance of the thalamus in the functional connectome in childhood focal epilepsies, particular in extratemporal epilepsy which has recently been reported to constitute 37.9% of all pediatric epilepsy surgeries (21). We therefore set out to test the hypothesis that the anterior nucleus of the thalamus has a higher level of functional connectivity in children with focal epilepsy than in those without epilepsy. We aimed to do this by using graph theory to measure connectiveness (degree and eigenvector centrality) of the anterior thalamus, as performed in prior studies (16). We additionally wished to test the hypothesis that these changes are not predominantly due to transient effects, but rather persisting alterations in the network.

## Methods

**Figure 1:**
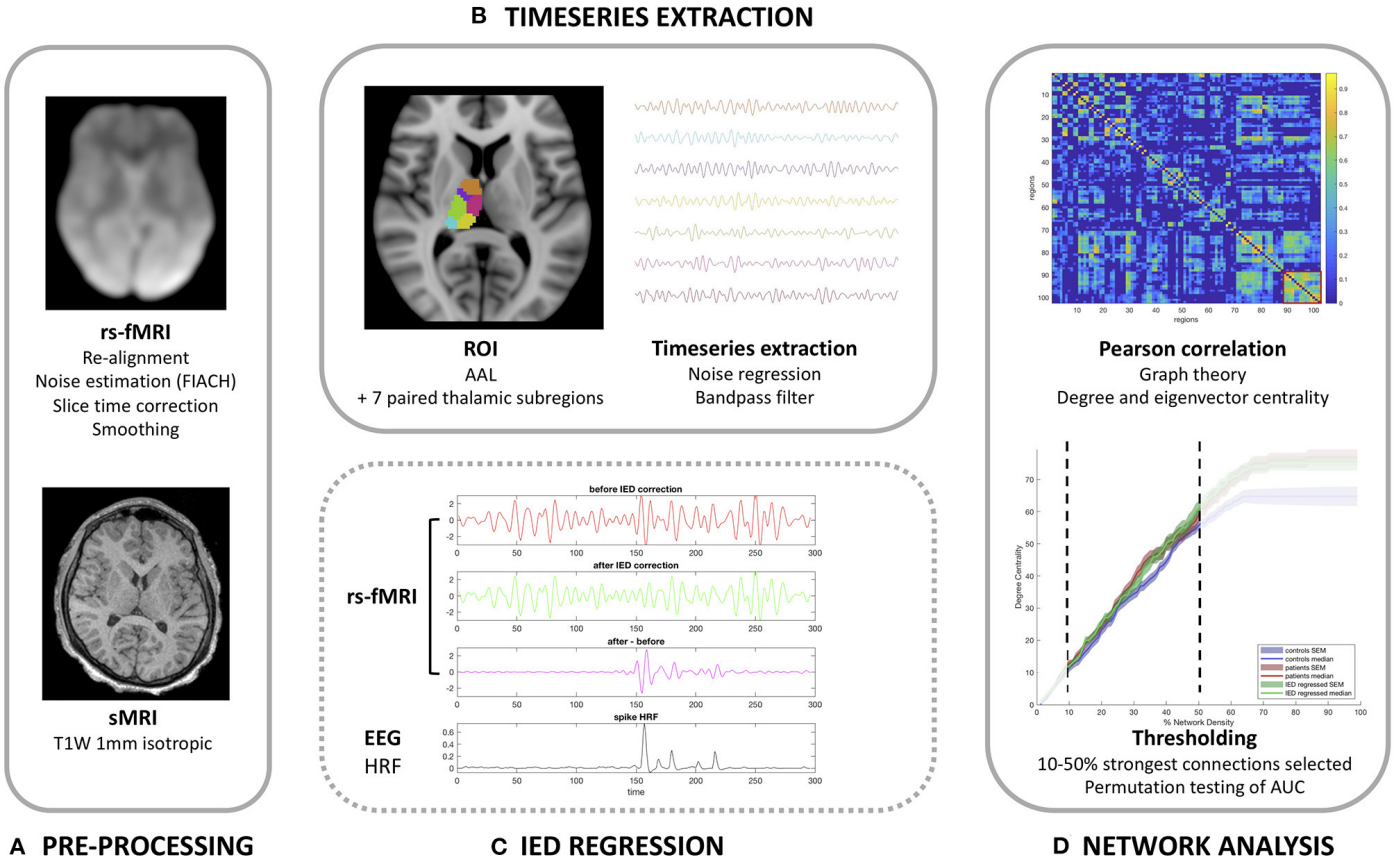
Imaging analysis pipeline. **(A)** Pre-processing pipeline. **(B)** Timeseries extraction. **(C)** Demonstration of the correction method for interictal epileptiform discharges (IEDs) in the anterior nucleus of the thalamus for patients 13 (one spike type) as detailed in Table 1. **(D)** Functional network analysis.

Our methodology and imaging pipeline is summarized in Figure 1.

**Table 1:** Demographic, clinical, neurophysiological, and neuroradiological descriptions of each child.

**ID**	Age^*^ (years)	Age of onset^**^ (years)	**Sex**	**Laterality of EZ**	**Location of EZ**	**MRI features**	**Focal vs. multifocal**	**No. of IEDs**	**Medications**
1	8		Female	Left	Temporal	Tuberous sclerosis	Focal	2	NZP and ZNS
2	14	4	Female	Left	Frontal	Cryptogenic	Focal	2	LCM and LVT
3	11	0.25	Male	Left	Hypothalamus/temporal	Hypothalamic hamartoma	Focal	59	LVT
4	15	10	Male	Left	Posterior quadrant	Cryptogenic	Multifocal	31, 10	CBZ
5	17		Male	Right	Parietal	Focal cortical dysplasia	Multifocal	51, 37, 71, 28	LVT, CBZ, and VPA
6	15	10	Male	Right	Frontal-central	Cryptogenic	Focal	15	CBZ
7	17	3	Female	Left	Temporal	Cryptogenic	Multifocal	175, 30, 131	LVT
8	14	2.5	Female	Right	Temporal	Focal cortical dysplasia	Multifocal	206, 16	LVT and TOP
9	11	6	Female	Right	Frontal-temporal	Cryptogenic	Focal	132	CBZ and LTG
10	11	7	Female	Right	Frontal	Focal cortical dysplasia	Focal	141	OXC
11	17	10	Female	Right	Frontal	Cryptogenic	Focal	34	LTG and LEV
12	16	6	Female	Left	Frontal	Cryptogenic	Focal	7	VPA and CBZ
13	16	13	Female	Left	Insula	Focal cortical dysplasia	Focal	76	TPM and CBZ
14	11	3	Male	Right	Frontal	Cryptogenic	Multifocal	128, 29	CBZ
15	11	8	Male	Right	Frontal	Cryptogenic	Multifocal	25, 1	LVT and VPA
16	16	2	Female	Left	Temporal-parietal-occipital	Polymicrogyria	Multifocal	236, 62, 39	LEV and CLNZ
17	15	9	Male	Left	Temporo-occipital	Hippocampal sclerosis	Multifocal	83, 21	LMT 575 Zonasimne 200
18	15	8	Male	Left	Fronto-temporal	Focal cortical dysplasia	Focal	112	OXZC 1200 LVT
19	17	5	Female	Left	Frontal	Cryptogenic	Focal	82	OXC
20	8	4	Female	Left	Frontal	Middle cerebral artery stroke	Focal	150	VPA 1200, LEV 600, ETHX 1000
21	16		Male	Left	Frontal	Cryptogenic	Multifocal	129, 77	PMP
22	13	3	Male	Left	Frontal	Focal cortical dysplasia	Focal	26	OXC and CLBZ
23	10	3	Female	Left	Frontal	Cryptogenic	Multifocal	47, 4	LVT and CBZ
24	11	6	Male	Right	Posterior quadrant	Cryptogenic	Multifocal	25, 23, 5	OXC
25	17	8	Male	Left	Frontal	Cryptogenic	Multifocal	35, 13	LVT, OXC, and CLBZ
26	17		Male	Right	Occipital	Ischaemic perinatal insult	Focal	21	OXC, LMT, and LVT 2000
27	18		Female	Right	Frontal	Focal cortical dysplasia	Multifocal	148, 6	LTG
28	17	5	Female	Right	Fronto-temporal	Bilateral polymicrogyria	Multifocal	242, 43	LVT and VPA
29	11		Female	Right	Parietal	Cryptogenic	Focal	81	OXC, PHE, and CLBZ
30	17	5	Male	Left	Frontal	Cryptogenic	Focal	67	LVT and LAC
31	11	5	Female	Right	Parietal	Focal cortical dysplasia	Focal	450	OXC, CLBZ, and VPA
32	17	12	Female	Left	Frontal	Cryptogenic	Multifocal	62, 1, 75	VPA
33	13	7	Female	Left	Frontal	Focal cortical dysplasia	Multifocal	128, 23	VPA
34	15		Female	Right	Posterior cingulate	Dysembryoplastic neuroepithelial tumor	Focal	26	LVT and LTG
35	7		Male	Left	Frontal	Cryptogenic	Focal	145	LVT, OXC, and CLBZ

*The number of interictal epileptiform discharges (IEDs) are listed for each IED type within each patient. EZ, epileptogenic zone; IED, interictal epileptiform discharge;*

* *Age rounded to nearest integer*.

** *Age-of-onset (if known) is an approximate. CBZ, Carbamazepine; CLBZ, Clobazam; GAB, Gabapentin; LCM, Lacosamide; LTG, Lamotrigine; LVT, Levetiracetam; NZP, nitrazepam; OXC, Oxcarbazepine; PHE, Phenobarbital; PGB, Pregabalin; PMP, Perampanel; RUF, Rufinamide; TPM, Topiramate; VPA, Valproate; ZNS, Zonisamide*.

### Patients

This study examined data obtained from 35 children with drug-resistant, focal epilepsy undergoing evaluation for epilepsy surgery at Great Ormond Street Hospital (London, United Kingdom) (20/35 female, median age 15 years, age range 7–18 years), previously described in the work by Centeno et al. (19, 22). We excluded patients in whom there were large lesions that deformed brain architecture to ensure a reliable parcellation of brain regions would be possible in all subjects. The putative epileptogenic zone (determined by the epilepsy multidisciplinary team by means of clinical, neurophysiological, and neuroimaging data) was most commonly the frontal lobe (21/35 cases). The putative epileptogenic zone was lateralized to the left-side in 20/35 children. We summarize the patient details individually in Table 1 and at a group level in Table 2. Our control group consisted of twenty children without epilepsy (13/20 female, median age 10 years, age range 7–17 years). Ethical approval was given by the NRES Committee London: Surrey Borders Research Ethics Committee London Center (REC reference: 11/LO/1421). The guardian of each participant provided informed and written consent on the participant's behalf.

**Table 2:** Summaries of the patient and control cohorts.

	**Patients**	**Controls**
Median age (range)	15 (7–18) years	10 (7–17) years
Male:female ratio	15:20	7:13
Median age of seizure onset (range)^*^	6 (0.25–13) years	–
Median duration of epilepsy^*^	7 (3–14) years	–

* *Data missing for eight patients*.

### Magnetic Resonance Imaging Acquisition

All subjects underwent simultaneous EEG-fMRI according to the protocol previously published Centeno et al. (22). Briefly, subjects were scanned at Great Ormond Street Hospital (London, United Kingdom) in a 1.5T Siemens Avanto scanner using a 12 channel receive head coil. One cubic millimeter isotropic T1-weighted images were acquired using a Fast Low Angle Single Shot (FLASH) gradient echo sequence. The fMRI acquisition consisted of echo planar imaging (EPI) with 3.3 × 3.3 × 4 mm resolution with a field of view of 210 mm, TR of 2,160 ms, TE of 30 ms and flip angle of 75. There were 30 contiguous slices in each volume with a slice thickness of 3 mm, slice gap of 1 mm, and matrix of 64 × 64. There were 300 volumes per session but the first five volumes in each session were omitted. Each subject underwent up-to four fMRI sessions (each for 10 min and 48 s), based on their ability to tolerate all of the sessions. In two randomly allocated sessions the child watched a cartoon, and in the other two sessions the child was asked to rest with their eyes closed. We chose to include the first resting fMRI session, in which children were asked to rest with their eyes closed, since this session had reduced motion compared to the second on average (22).

### Image Pre-processing

Image pre-processing was performed using Statistical Parametric Mapping (SPM; https://www.fil.ion.ucl.ac.uk/spm/software/spm12/). fMRI data was re-aligned and then the Functional Image Artifact Correction Heuristic (FIACH) method (23) was applied. This removes biophysically implausible signal jumps, and creates a parsimonious noise model from brain regions exhibiting high noise levels. This method has been shown to be highly effective when compared to a number of alternative pipelines (23, 24). The six noise regressors estimated by FIACH were added to the six re-alignment parameters generated by SPM. The fMRI data was then corrected for slice-timing and then registered to the 1 mm isotropic T1W volume. Image registration was visually verified for each subject. Each patient's T1W volumes were normalized to standard space (MNI 152 template) and then the fMRI was normalized using the derived transform. The fMRI volumes were then converted to a 2 mm isotropic resolution following the normalization step. A Gaussian kernel was used to smooth fMRI 8 mm in each direction.

### Electroencephalography (EEG) Acquisition and Analysis

Simultaneous scalp EEG data was acquired using a 64-channel, MRI-compatible cap (*EASYCAP*, Brain Products, Munich, Germany). The full EEG acquisition protocol is available in prior work (22). The onsets and durations of IEDs for each session were identified by a neurologist and neurophysiologist, as described in prior work (19, 22). Events onsets and durations were used to generate a temporal regressor by convolution with the standard canonical hemodynamic response, and its temporal and dispersion derivatives as implemented in SPM8.

### Timeseries Analyses

Determination of the fMRI timeseries for each region within the brain had the following processing steps applied using MATLAB (MathWorks, Natick, MA, United States). This script is freely available to download (https://github.com/roryjpiper/rs-fMRI.git). A general linear model was applied to control noise according to the six re-alignment parameters aforementioned. We then took the mean timeseries signal from the voxels of each region of the brain according to the Automated Anatomical Labeling (AAL) template (25). The cerebellar regions were excluded to leave 90 cerebral regions. We investigated thalamic nuclei by removing the left and right thalami from the AAL atlas and replaced these with seven paired thalamic subregions, which instead parcellated the cerebrum into 102 regions. The sub-parcellations of the thalamus used here are described in previously published work by He et al. (26). These thalamic subregions included the anterior, medial dorsal, lateral dorsal, lateral posterior, ventral lateral posterior, medial pulvinar and lateral pulvinar subregions (shown in Figure 1). The timeseries signal for each region was then band-pass filtered to 0.04–0.07 Hz as per our previous work (27).

### Correction for the Effects of Inter-ictal Epileptiform Discharges

**Figure 2:**
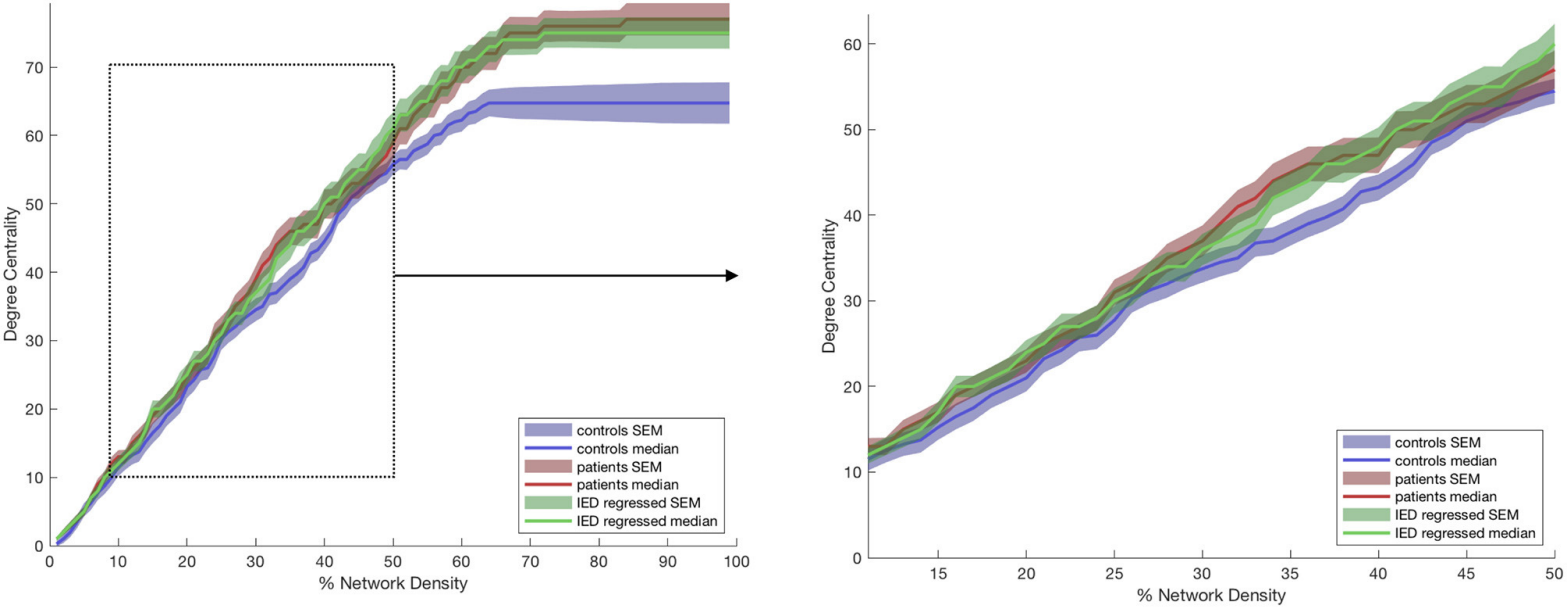
Degree centrality of the anterior thalamic subregion ipsilateral to the epileptogenic zone (EZ). The lines represent controls (blue), patients before (red) and after (green) correction for interictal epileptiform discharges (IEDs). The left graphs show the full range of network densities from 0 to 100%. The dotted box shows the range selected for statistical analysis and shown in the right graph.

Following the procedure described in detail by Shamshiri et al. (20), we used functions in R (Version 3.6.1) to remove the influence of IEDs from the fMRI signal. Figure 2 demonstrates the effect of IED regression on fMRI signal. Briefly, IED signal changes are modeled by convolving the IEDs with the canonical hemodynamic response function, and its derivatives before projecting the data from each region into an orthogonal space. Following this correction, the “IED-corrected” timeseries' for each patient was determined using the identical pipeline as described above.

### Graph Theory Analyses

We created a 102 × 102 adjacency matrix for each subject by calculating the Pearson correlation coefficient between each region using the *corcoeff.m* MATLAB function. We re-assigned the values in the diagonal of the adjacency matrix (self-correlation) to 0. All negative correlation values were re-assigned to 0. We used the Brain Connectivity Toolbox (29) (www.brain-connectivity-toolbox.net) in MATLAB to calculate the degree centrality (DC) (number of links connected to a node) and eigenvector centrality (EC) (self-referential measure of a node influence) for each region-of-interest (ROI) in every individual. These measures were selected to allow us to interpret our findings in the context of prior work in thalamic connectivity (16), but also are selected to test our hypothesis that brain activity will have greater synchrony in children with epilepsy when compared to those without.

### Statistical Analyses

For the purpose of statistical analysis, we selected a range of 10–50% of the highest correlations found in the adjacency matrix. Fifty percent was used as the upper limit since after this point the number of connections (defined as positive correlation values) did not increase any further in some subjects. To determine the difference in DC and EC between groups that are relatively robust to our choice of network density and thresholds, we compared results derived from the thalamus ipsilateral to hemisphere of the epileptogenic focus in patients to the median results derived from the left and right thalami of controls. Using a MATLAB-based permutation test (30), we statistically compared the median area-under-the-curve (AUC) between 10 and 50% of the network density for each graph theory metric (see Figure 2) between the results for these subject groups. Statistical testing for measures of the anterior nucleus of the thalamus were performed with an established priori hypothesis, whereas those performed on the remaining thalamic subregions were exploratory, and the reported *p*-values should be interpreted as descriptive. Our statistical testing used 10,000 permutations and outputted a two-tailed *p*-value. The effect of IEDs on the graph theory measures were further examined by measuring the Spearman correlation of the residual AUC for each patient vs. the number of spikes per session. Effect size was determined using the Mann-Whitney-Wilcoxon test (*r* = *z* / √ *n*_1_ + *n*_2_). Figure representation of data uses the median value ± standard error of the mean (SEM). Decimal places are rounded to two decimal points.

## Results

### Degree and Eigenvector Centrality

**Figure 3:**
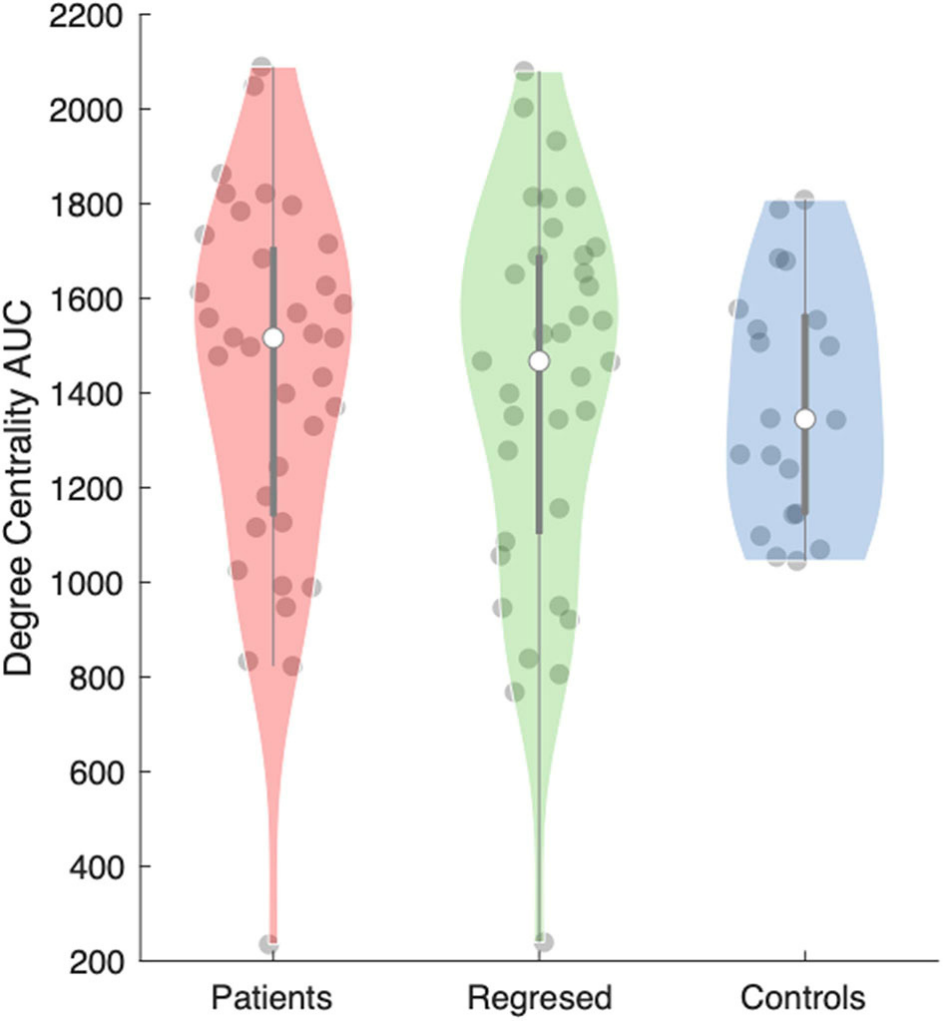
Area under the curve (AUC) for the degree centrality of the anterior thalamic subregion ipsilateral to the epileptogenic zone (EZ). The violin plot (28) demonstrates the distributions of the AUC for degree centrality in patients before (red) and after (green) interictal epileptiform discharges (IEDs) and controls (blue). The clear dot shows the median.

The anterior nucleus of the thalamus ipsilateral to the EZ was found to have a significantly higher AUC for DC in children (1,516.5 ± 66.15) with epilepsy compared to controls (1,345 ± 56.41) (*r* = 0.10; *p* = 0.04) (Figures 2, 3). We performed the same analysis on only the patients with a frontal EZ (*n* = 17) and found a similar trend in DC, but this did not reach statistical significance (*p* = 0.38).

We then performed an exploratory analysis of the medial dorsal, lateral dorsal, lateral posterior, ventral lateral posterior, medial pulvinar and lateral pulvinar thalamic subregions, on comparing these two groups again, the median AUC for DC was also significantly higher in the ipsilateral lateral dorsal nucleus (*p* = 0.02) (Supplementary Figure 1).

No significant difference was found, however, for the same analyses for the ipsilateral thalamus, taken as a whole (the AAL thalamus ROI) (*p* = 0.30), nor for any of the remaining five nuclei (Supplementary Figure 2). No significant differences were seen in EC between patients and controls (Supplementary Figure 2).

### Effects of Interictal Epileptiform Activity

Total number of captured IEDs ranged from 2 to 450 per EEG-fMRI session, as detailed in Table 1. Nineteen patients had one spike type (focal) and 16 had more than one IED type. We did not detect significant differences in the median AUC for DC (*p* = 0.62) or EC (*p* = 0.81) in the anterior thalamic nucleus when comparing between the results from children with epilepsy before, and after the effects of IEDs were removed. No other thalamic subregion showed these differences either.

When correlating between IED frequency and the AUC for DC in the ipsilateral anterior nucleus in children with epilepsy, we found a borderline, but non-significant negative correlation in DC [ρ = −0.34; *p* = 0.05 (rounded up to two decimal places)], and a significant negative correlation in EC (ρ = −0.38; *p* = 0.03).

## Discussion

The anterior nucleus of the thalamus has consistently been an area of clinical interest and a therapeutic target in patients with epilepsy. High-frequency stimulation of the anterior nucleus has been shown to desynchronize focal large-scale brain activity and reduce the number of IEDs in adults with TLE (31). The SANTE trial in 2010 is, to date, the largest randomized controlled trial of stimulation of the anterior nucleus of the thalamus significant reduction in seizure frequency for adults with both TLE and those with epileptogenic foci elsewhere (14).

Our study suggests that the number of connections (degree centrality [DC]), determined by fMRI, is higher in the anterior nucleus of the thalamus ipsilateral to the EZ in children with focal epilepsy when compared to age-matched controls. Our findings support the significance of these nuclei in the functional connectome of children with epilepsy, and add weight to the hypothesis that DBS to these regions could be therapeutic.

The increased functional connectivity that we demonstrated in the anterior nucleus is in keeping with the aforementioned work, and also with a number of studies which have found the “midline thalamus” to be a key region involved in thalamo-temporal networks (32, 33). He et al. used resting state fMRI to study functional connectivity, and suggest that presurgical thalamic “hubness” (the thalamus being an important node in the network) is a biomarker for predicting seizure outcome in patients undergoing surgery for TLE (16). In patients not rendered seizure free, increased nodal hubness was seen in both the ipsilateral and contralateral thalami on account of an increased DC and EC. Furthermore, simulated lesioning of the thalami showed a greater reduction in network integration in the patients not rendered seizure free. In another study, Bonilha et al. (34), showed that a feature of the connectome after temporal lobectomy was reduced connectivity in thalamo-cortical circuits, and that patients were more likely to achieve seizure freedom if their preoperative network did not involve abnormal thalamic connectivity (35).

Furthermore, on exploratory analysis, we identified a higher functional connectivity of the lateral dorsal nucleus of the thalamus, but this in the context of a predominantly extra-temporal epilepsy cohort. The lateral dorsal nucleus has been shown to have connections with the limbic lobe (including cingulate gyrus), parietal and visual cortex (36). Furthermore, it has been postulated that the anterior and lateral dorsal nuclei, may represent a “higher order” set of thalamic nuclei that, rather than act as a “simple relay,” have a more significant influence in the regulation of cortical-cortical interactions (37). It is important to note the limited spatial resolution and effects of co-registration that affect fMRI. It is possible that some of the effect seen in the lateral dorsal nucleus is a spillover of signal detected from the anterior nucleus.

The differences in functional connectivity we observed in this study, however, were of modest magnitude. In contradistinction to relatively homogeneous TLE cohort studies, this study included patients with an epileptic focus in various brain regions which is representative of the surgical population in pediatric practice. Additionally, thalamic connectivity in this cohort needs to be measured against a background of developmental changes. While both of these factors could dilute the effect in childhood epilepsy, we demonstrate a significant effect confirming our primary hypothesis. Our analysis of the patients in this dataset may have combined and averaged the results of distinct groups of children who either do or do not have the thalamus, or its subregions, as regions of higher connectivity within the functional network. It would be interesting to continue this work in this cohort by studying whether or not increased connectivity of the anterior thalamus is predictive of clinical outcome in children undergoing DBS.

Our secondary hypothesis was that altered functional connectivity of the anterior thalamic nuclei was not due to transient effects of IEDs, but instead to stable alterations in the network. We did not detect a significant difference in DC values in this region in patients after the effects of IEDs were corrected for. This study adds to the ongoing discussion regarding the effect of IEDs in the functional connectivity of the brain and the role of the thalamus. It could be that long-term alterations in thalamic connectivity facilitate the spread of IEDs and that thalamic DBS reduces seizure frequency by inhibiting this pathway of connectivity. This idea is supported by a study by Yu et al. (31) aforementioned, that showed that bilateral anterior thalamic nucleus stimulation reduces IEDs. An alternative hypothesis is that focal IEDs are the cause of increased thalamic connectivity, but our study did not show a significant difference between DC and EC in patients before, and after the effects of IEDs were removed, suggesting that the anterior and lateral dorsal thalamic regions have an intrinsically altered baseline. This is in keeping with prior work that suggests that increased connectivity within epileptogenic networks survives the regression of IED effects (38). A study by Shamshiri et al. (20) showed that IEDs can have a pervasive yet transient effect on the brain's functional organization using a seed-to-voxel analysis during a low level attention task. This is compatible with our study findings, where the anterior nucleus of the thalamus may facilitate a permissive state of increased connectivity whereby IEDs (from different brain regions in this heterogeneous group) can affect the coherence of “active” networks.

We acknowledge the following limitations of our study. Firstly, our patient group was heterogeneous in etiology and epileptogenic focus (with a frontal lobe predominance). This could, however, be observed as a strength since the findings we have detected have survived in this mixed cohort, which is somewhat reflective of the pool of patients referred for epilepsy surgery workup. Secondly, the AAL atlas was designed for the analysis of the adult MNI-registered brain. Although we have visually validated the registration of the AAL atlas in each case and used a kernel smoothing method for fMRI signal, there may be inaccuracies that are unavoidable when using this brain region atlas in children. Due to limited sample size and heterogeneous surgical management, we could not make meaningful correlations of clinical outcomes with our quantitative graph theory metrics. Lastly, we recognize the need to validate these findings in external cohorts, particularly in children with generalized epilepsy or those with focal seizures with secondary generalization.

## Data Availability Statement

The raw data supporting the conclusions of this article will be made available by the authors, without undue reservation.

## Ethics Statement

The studies involving human participants were reviewed and approved by the NRES Committee London: Surrey Borders Research Ethics Committee London Center (REC reference: 11/LO/1421). Written informed consent to participate in this study was provided by the participants' legal guardian/next of kin.

## Author Contributions

All authors listed have made a substantial, direct and intellectual contribution to the work, and approved it for publication.

## Conflict of Interest

The authors declare that the research was conducted in the absence of any commercial or financial relationships that could be construed as a potential conflict of interest.

## Publisher's Note

All claims expressed in this article are solely those of the authors and do not necessarily represent those of their affiliated organizations, or those of the publisher, the editors and the reviewers. Any product that may be evaluated in this article, or claim that may be made by its manufacturer, is not guaranteed or endorsed by the publisher.
