# Supplementary material for: Functional Connectivity of the Anterior Nucleus of the Thalamus in Pediatric Focal Epilepsy
Source: Front Neurol. 2021 Aug 2;12:670881. doi: 10.3389/fneur.2021.670881 (PMC8365837; doi:10.3389/fneur.2021.670881)
Supplement: Supplementary file 1 [file Data_Sheet_1.docx]

Supplementary Material


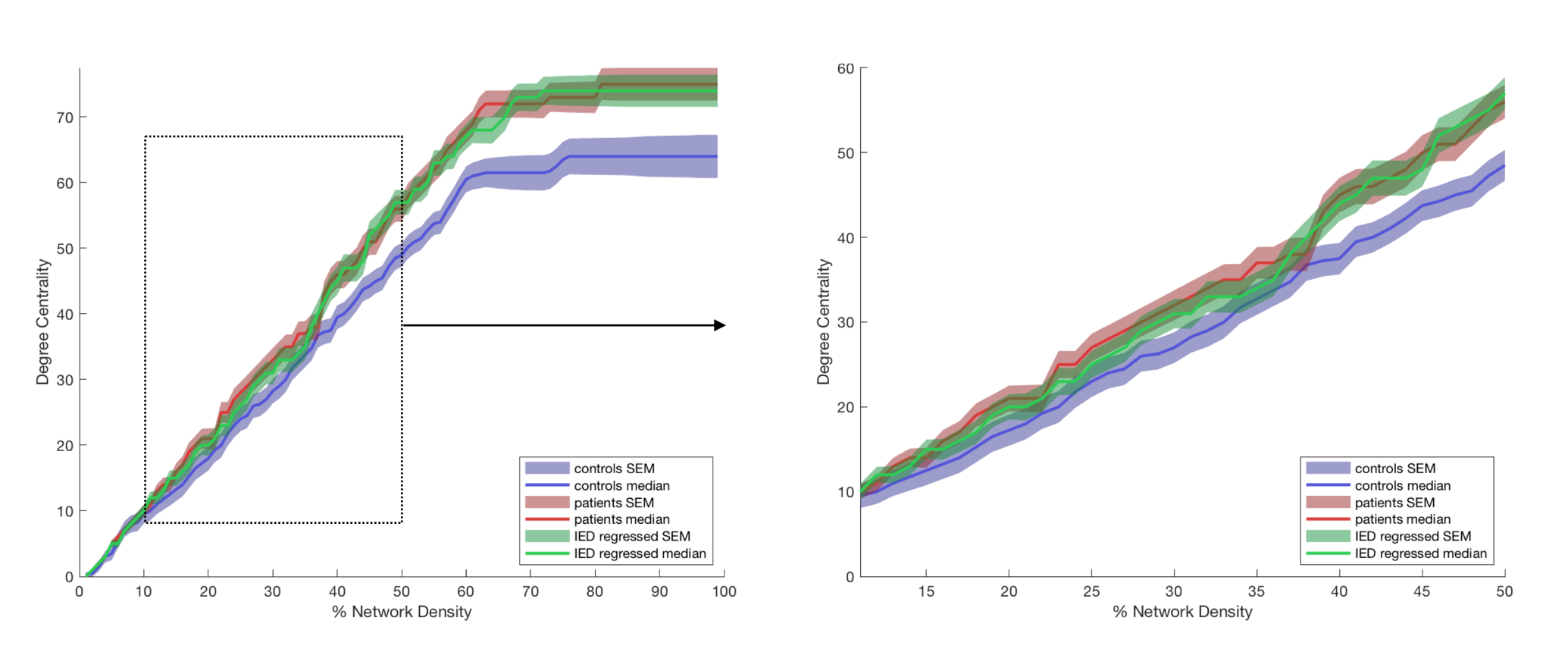


**Figure S1.** **Degree centrality of the lateral dorsal thalamic subregion ipsilateral to the epileptogenic zone (EZ).** The lines represent controls (blue), patients before (red) and after (green) correction for interictal epileptiform discharges (IEDs). The left graphs show the full range of network densities from 0% to 100%. The dotted box shows the range selected for statistical analysis and shown in the right graph.


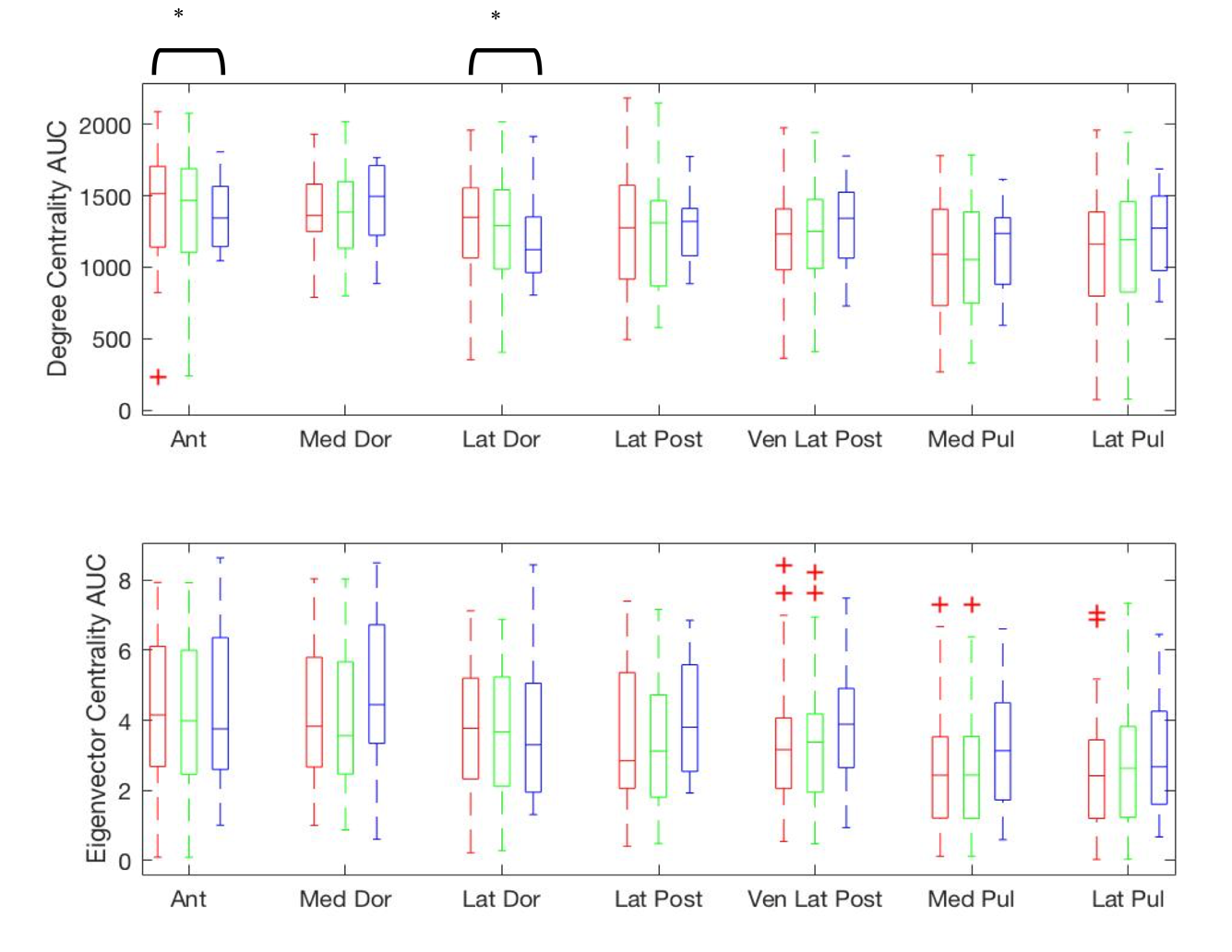


**Figure S2.** **Summary of graph theory measures per each thalamic subregion ipsilateral to the epileptogenic zone (EZ).** Degree (top) and eigenvector (bottom) centrality measures in patients (red), patients with the effects of interictal epileptiform discharges regressed out (green), and controls (blue). The median value and interquartile ranges are given for each subject group in each of the thalamic subregions: anterior (Ant), medial dorsal (Med Dor), lateral dorsal (Lat Dor), lateral posterior (Lat Post), ventral lateral posterior (Ven Lat Post), medial pulvinar (Med Pul), and lateral pulvinar (Lat Pul). ***** shows significant differences between the patient and control groups. + icons show the outliers.

**
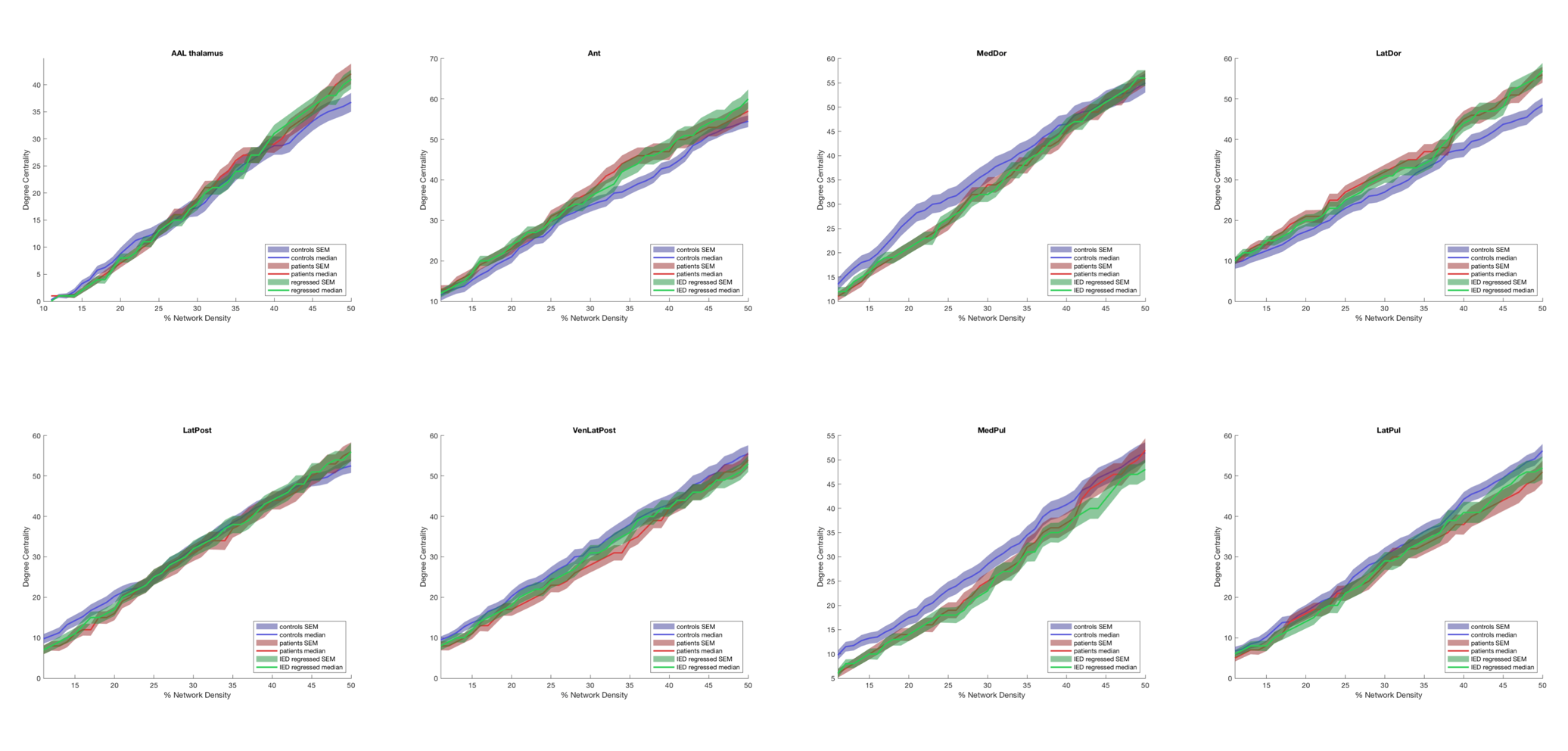
Figure S3.** Degree centrality for the AAL thalamus and thalamic subregions ipsilateral to the epileptogenic zone (EZ): anterior (Ant), medial dorsal (Med Dor), lateral dorsal (Lat Dor), lateral posterior (Lat Post), ventral lateral posterior (Ven Lat Post), medial pulvinar (Med Pul), and lateral pulvinar (Lat Pul).


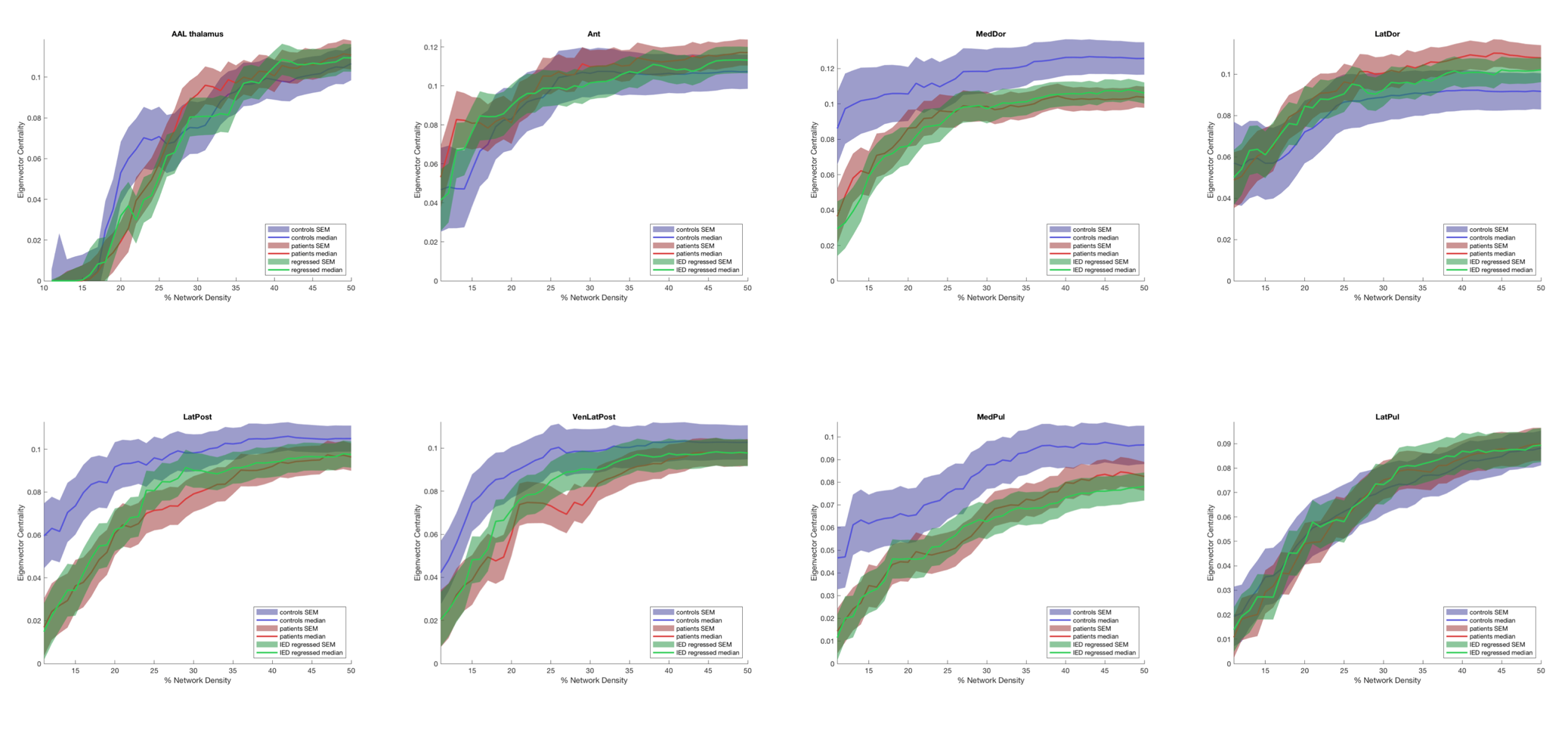


**Figure S4.** Eigenvector centrality for the AAL thalamus and thalamic subregions ipsilateral to the epileptogenic zone (EZ): anterior (Ant), medial dorsal (Med Dor), lateral dorsal (Lat Dor), lateral posterior (Lat Post), ventral lateral posterior (Ven Lat Post), medial pulvinar (Med Pul), and lateral pulvinar (Lat Pul).
